# The multifaceted role of vitamin B_6_ in cancer: *Drosophila* as a model system to investigate DNA damage

**DOI:** 10.1098/rsob.200034

**Published:** 2020-03-25

**Authors:** Roberto Contestabile, Martino Luigi di Salvo, Victoria Bunik, Angela Tramonti, Fiammetta Vernì

**Affiliations:** 1 Istituto Pasteur Italia-Fondazione Cenci Bolognetti and Dipartimento di Scienze Biochimiche ‘A. Rossi Fanelli’, Sapienza Università di Roma, P.le A. Moro, 5, 00185, Roma, Italy; 2 Belozersky Institute of Physico-Chemical Biology, Lomonosov Moscow State University, Moscow 119991, Russia; 3 Faculty of Bioengineering and Bioinformatics, Lomonosov Moscow State University, Moscow 119991, Russia; 4 Sechenov Medical University, Sechenov University, 119048 Moscow, Russia; 5 Istituto di Biologia e Patologia Molecolari, Consiglio Nazionale delle Ricerche, Pl.e A. Moro, 5, 00185 Roma, Italy; 6 Dipartimento di Biologia e Biotecnologie ‘Charles Darwin’, Sapienza Università di Roma, Pl.e A. Moro, 5, 00185 Roma, Italy

**Keywords:** vitamin B_6_, genome integrity, cancer, *Drosophila melanogaster*

## Abstract

A perturbed uptake of micronutrients, such as minerals and vitamins, impacts on different human diseases, including cancer and neurological disorders. Several data converge towards a crucial role played by many micronutrients in genome integrity maintenance and in the establishment of a correct DNA methylation pattern. Failure in the proper accomplishment of these processes accelerates senescence and increases the risk of developing cancer, by promoting the formation of chromosome aberrations and deregulating the expression of oncogenes. Here, the main recent evidence regarding the impact of some B vitamins on DNA damage and cancer is summarized, providing an integrated and updated analysis, mainly centred on vitamin B_6_. In many cases, it is difficult to finely predict the optimal vitamin rate that is able to protect against DNA damage, as this can be influenced by a given individual's genotype. For this purpose, a precious resort is represented by model organisms which allow limitations imposed by more complex systems to be overcome. In this review, we show that *Drosophila* can be a useful model to deeply understand mechanisms underlying the relationship between vitamin B_6_ and genome integrity.

## 1. Impact of most representative B group vitamins on DNA damage and cancer: *in vitro* and *in vivo* studies

The study of micronutrients is a topic of general interest, due to the impact of minerals and vitamins on human health. Growing evidence shows that the deficiency of several vitamins causes DNA damage predisposing to cancer and neurological diseases, but cause–effect relationships in most of the cases are not completely understood. Many micronutrients work as cofactors or substrates for enzymes that counteract genotoxins or are involved in DNA metabolism, and their deficiency can damage DNA analogously to common carcinogens [1]. In many cases, it is difficult to finely predict the optimal rate of micronutrients that is able to protect against DNA damage, as this rate can be influenced by the individual's genotype [2]. Thus, the need arises to explore in depth the pleiotropic action and the metabolism of vitamins, in order to set supportive interventions and personalized cares.

**Figure 1.**
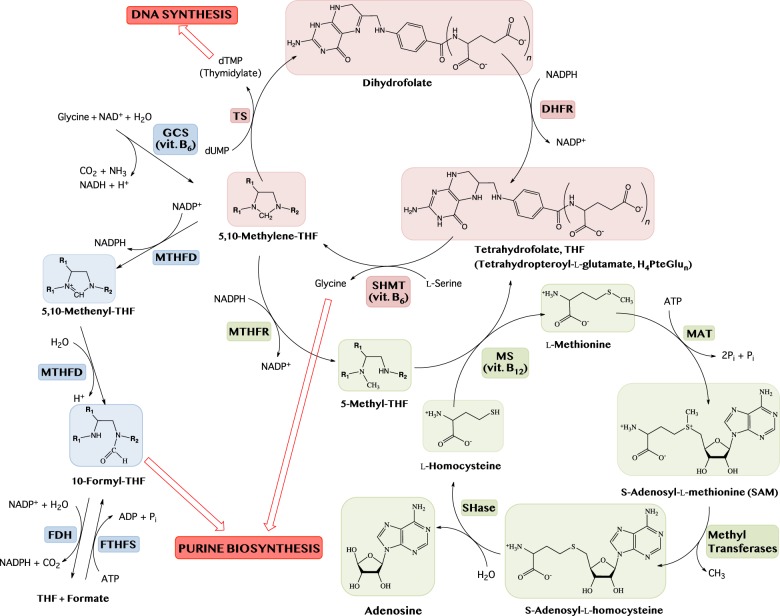
Schematic of B_9_ metabolism comprising the thymidylate cycle (red diagram), the methionine cycle (green diagram) and the purine biosynthesis pathway (blue diagram). The enzymes involved are: dihydrofolate reductase (DHFR); thymidylate synthase (TS); serine hydroxymethyltransferase (SHMT); methylenetetrahydrofolate reductase (MTHFR); methionine synthase (MS); methionine adenosyltransferases (MAT); *S*-adenosylhomocysteinase (SHase); glycine cleavage system (GCS); methylenetetrahydrofolate dehydrogenase (MTHFD); 10-formyltetrahydrofolate dehydrogenase (FDH); formyltetrahydrofolate synthetase (FTHFS).

Vitamins B_9_, B_12_, B_1_ and B_6_ (dietary sources reported in table 1) are the source of coenzymes that participate in one carbon metabolism, in which 1C units are used in biosynthetic processes such as purine and thymidylate synthesis and homocysteine remethylation (figure 1). Consistently, a large body of evidence shows that deficiency of these vitamins impacts on genome stability and cancer. Vitamin B_9_ encompasses a group of compounds collectively named as folates, including folic acid, tetrahydrofolic acid (THF; or H_4_-pteroyl-L-glutamate), 5-methyltetrahydrofolic acid (CH_3_-THF) and 5,10-methylenetrahydrofolic acid (CH_2_-THF), required for growth and development. Dietary folic acid is first reduced to dihydrofolate and then to tetrahydrofolate by the activity of dihydrofolate reductase. Folate deficiency (FD) causes genome instability as assessed by *in vitro* studies on human and animal cell cultures. In particular, FD produces fragile sites [3], chromosome breakage [4] and aneuploidy [5]. Cytokinesis-block micronucleus assays in primary human lymphocyte cultures deprived of folate revealed micronuclei, which contain chromosomes or chromosome fragments not incorporated into one of the daughter nuclei during cell division, nucleoplasmic bridges (a biomarker of dicentric chromosomes resulting from telomere end-fusions or DNA misrepair) and nuclear buds (a marker of gene amplification and/or altered gene dosage) [6].

**Table 1.** Dietary sources and recommended daily allowance for vitamins B_1_, B_6_, B_9_ and B_12_ (from https://www.ncbi.nlm.nih.gov/books/NBK554545/).

vitamin	dietary sources	RDA (recommended dietary allowance)
vitamin B_1_ (thiamine)	found in all foods in moderate amounts: pork, legumes, enriched and whole grains, cereals, nuts and seeds	1.1 mg day^−1^ for adult women and 1.2 mg day^−1^ for adult men
vitamin B_6_ (pyridoxine)	widespread among food groups: meat, fish, poultry, vegetables, fruits	1.3 mg day^−1^ for adults
vitamin B_9_ (folic acid)	leafy green vegetables and legumes, liver, seeds, orange juice, enriched and fortified grains	400 mcg day^−1^ of *dietary folate equivalents*^a^ for adults; recommendation is that women of childbearing age consume an additional 400 mcg day^−1^ of folic acid from supplements or fortified foods to decrease the risk of neural tube defects
vitamin B_12_ (cobalamin)	only present in animal products because it is a product of bacteria synthesis: meat, poultry, fish, seafood, eggs, milk and milk products; not found in plant foods; many foods are also fortified with synthetic vitamin B_12_	2.4 mcg day^−1^ for adults; it is recommended for older adults to meet their RDA with fortified foods or supplements because many are unable to absorb naturally occurring vitamin B_12_

^a^Dietary folate equivalents (DFE) take into account the lower availability of mixed folates in food compared with synthetic tetrahydrofolate used in food enrichment and supplements. Currently, the use of DFE is recommended for planning and evaluating the adequacy of people's folate intake.

*In vitro* observations have been complemented with epidemiological [7,8] and controlled intervention studies [9–11], further reinforcing the association between folate and genome stability. Consistently, a growing body of evidence indicates that FD may increase risk for several cancer, including those of colon, pancreas, prostate and breast [12,13]. To explain the effects of FD on genome stability, two mechanisms have been proposed: the impaired conversion of dUMP in dTMP and the hypomethylation of DNA. Folate is required for conversion of deoxyuridine monophosphate (dUMP) to deoxythymidine monophosphate (dTMP) performed by thymidylate synthase (TS) (figure 1). Therefore, FD can cause dUTP incorporation in DNA, instead of dTTP, which is removed by uracil glycosidase, resulting in mutations, chromosome aberrations and eventually cancer. In addition, the unbalanced dUTP/dTTP ratio can impair DNA synthesis and repair, increasing genetic instability. As a confirmation of this model, treatment of human lymphoid cells in culture with methotrexate, an inhibitor of dihydrofolate reductase, increases the dUTP/dTTP ratio and the rate of uracil misincorporation in DNA [14]. Moreover, *in vitro* folic acid depletion causes uracil misincorporation in human lymphocytes [15].

Folate is also required for the production of *S*-adenosylmethionine (SAM) throughout the remethylation of homocysteine to methionine (figure 1). In turn, SAM regulates gene transcription by methylating specific cytosines in DNA. As a consequence, low folate levels may lead to DNA hypomethylation, which can potentially induce proto-oncogenes expression. An altered methylation pattern has been proposed to be at the basis of aneuploidy caused by FD. According to this model, it has been proposed that demethylation of heterochromatic centromeric regions could impair the correct distribution of chromosomes during nuclear division [16]. However, more recently, it has been proposed that aneuploidy in FD cells can also result from spindle assembly checkpoint (SAC) dysfunction, due to an altered expression of some SAC genes induced by FD [17].

Vitamin B_12_ is essential for maintaining nervous system functions as well as haematopoiesis [18,19]. Suboptimal B_12_ status (serum B_12_ < 300 pmol l^−1^) is very common, occurring in 30–60% of the population, in particular in pregnant women and in less-developed countries. Vitamin B_12_, together with folate, serves as coenzyme for methionine synthase (MS) (figure 1). When B_12_ is insufficient, THF is trapped as CH_3_-THF. This hinders the regeneration of THF and reduces the size of the CH_2_-THF pool, leading to increased dUTP misincorporation into DNA. Reduced MTR activity increases homocysteine in tissue and plasma, a biomarker associated with several diseases, including risk of neural tube defects [20].

The first evidence that vitamin B_12_ deficiency is associated with chromosome damage in human cells has been the presence of ‘Howell–Jolly bodies’ in erythrocytes from patients affected by megaloblastic anaemia (a disease caused by B_12_ deficiency). Howell–Jolly bodies are small round nuclear remnants caused by chromosome breakage and chromosome segregation defects, as they contain whole chromosomes or chromosome fragments that lag behind at anaphase [21]. In line with these findings, subsequent *in vivo* and *in vitro* studies have associated low B_12_ levels with increased chromosome damage, and a significant negative correlation has been demonstrated between micronucleus index and serum vitamin B_12_ content [9,22–24]. Intervention studies showed that DNA damage and micronucleus frequency is significantly improved through vitamin B_12_ therapy [23,25,26].

Although low B_12_ levels are also expected to be associated with cancer, there is only little evidence on this. Indirect evidence come from a study indicating that smokers with low B_12_ levels had high rate of micronuclei, suggesting that low B_12_ levels could be correlated to epithelial cancers [27]. Another study suggested that elevated total B_12_ could be considered as a potential marker for oncohaematological disorders [28].

The coenzyme active form derivative of vitamin B_1_ (thiamine) is thiamine pyrophosphate (TPP), an essential cofactor of several key enzymes in cellular metabolism, among which is transketolase (TKT) within the pentose phosphate pathway (PPP). Three other phosphorylated forms have been observed intracellularly in humans in addition to TPP: thiamine monophosphate, thiamine triphosphate and adenosine thiamine triphosphate [29]. In cancer cells, TKT within the PPP is responsible for the synthesis of most ribose 5-phosphate (R5P). In normal cells, R5P is produced through the non-thiamine-dependent oxidative branch of PPP. If an excess of R5P is present with respect to cell requirements, it is recycled into glucose 6-phosphate through the non-oxidative branch of the PPP, in which TKT is present, where R5P is converted to fructose 6-phosphate and glyceraldehyde 3-phosphate. In cancer cells, the large requirement of R5P needed for nucleotide synthesis determines an inversion of the normal metabolic flux, increasing reliance on the non-oxidative branch PPP for R5P production [30]. Accordingly, inhibition of thiamine metabolism is expected to result in the reduction in the nucleotide pools. The thiamine analogue and anti-coenzyme oxythiamine was shown to reduce DNA and RNA synthesis, through reduction in R5P, and therefore tumour cell growth both *in vivo* and *in vitro* [31]. High importance of thiamine in malignant cells was shown in both epidemiological [32] and biochemical [33] studies. In humans, cancer rates correlate with thiamine status [32]. Thiamine depletion of normal tissues due to strong thiamine mobilization by cancer cells [33] may often cause complications in cancer patients, such as heart failure [34].

Thiamine and TPP have also been demonstrated to have antioxidant properties, reacting with ROS [35]. In particular, TPP has provided a greater protective effect against oxidative stress-induced damage (i.e. DNA hydroxylation) compared with thiamine [36].

## 2. Roles of vitamin B_6_ in human health and disease

### 2.1. Vitamin B_6_ metabolism in humans

**Figure 2.**
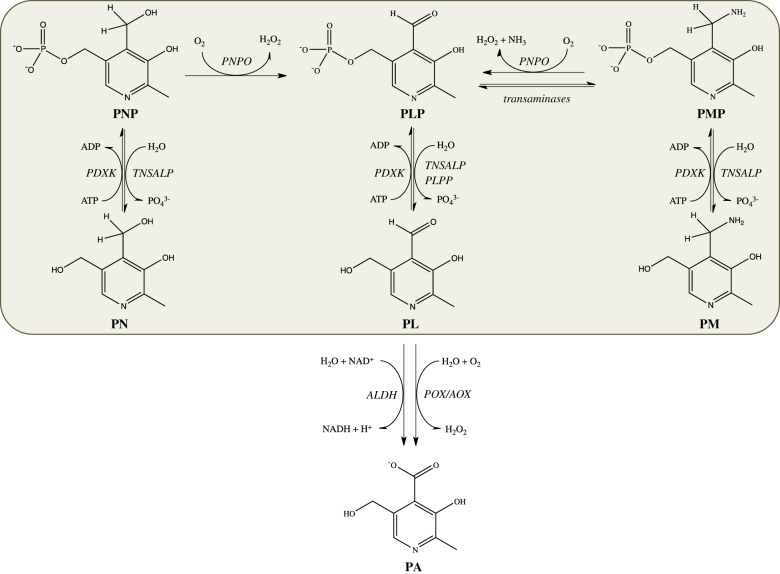
Schematic of vitamin B_6_ metabolism in humans. The orange diagram corresponds to the pyridoxal 5′-phosphate salvage pathway. PLP, pyridoxal 5'-phosphate; PNP, pyridoxine 5'-phosphate; PMP, pyridoxamine 5'-phosphate; PL, pyridoxal; PN, pyridoxine; PM, pyridoxamine; PA, 4-pyridoxic acid; PDXK: pyridoxal kinase; TNSALP: tissue-non-specific alkaline phosphatases; PLPP, pyridoxal 5'-phosphate phosphatase; ALDH, aldehyde dehydrogenases; POX, pyridoxal oxidase; AOX, aldehyde oxidases.

Vitamin B_6_ is an ensemble of six substituted pyridine compounds or vitamers: pyridoxine (PN), pyridoxal (PL), pyridoxamine (PM) and their related 5′-phosphate derivatives (figure 2). The catalytically active form of the vitamin, pyridoxal 5′-phosphate (PLP), acts as a cofactor for over 150 enzymes [37] involved in a number of crucial metabolic pathways, such as the synthesis, transformation and degradation of amines and amino acids, supply of one carbon units, transsulfuration, synthesis of tetrapyrrolic compounds (including haem) and polyamines, biosynthesis and degradation of neurotransmitters. Moreover, B_6_ vitamers are involved in important biological functions other than catalysis (table 2).

**Table 2.** Biological functions of B_6_ vitamers.

B_6_ vitamer	function	reference
PLP and PMP	catalysis (enzyme cofactor)	[37]
PLP and PN	binding to steroid receptors, playing a role in membrane transport	[38–40]
all vitamers	reactive oxygen species scavenger and resistance factor to biotic and abiotic stress in plants and in *Plasmodium falciparum*	[41–44]
PLP	virulence factor in *Helicobacter pylori*, *Mycobacterium tuberculosis* and *Actinobacillus pleuropneumoniae*	[45–47]
PLP	chaperone in enzyme folding	[48]
PLP	modulator of transcription factors	[49,50]
PLP and PMP	inhibition of the formation of advanced glycation end products (AGEs)	[51–53]

The different B_6_ vitamers are interconverted through a salvage pathway that involves pyridoxal kinase (PDXK), pyridoxine 5'-phosphate oxidase (PNPO) and several phosphatases (figure 2). The ATP-dependent PDXK phosphorylates the 5′ alcohol group of PN, PL and PM to form PNP, PLP and PMP, whereas the FMN-dependent PNPO oxidizes PNP and PMP to give PLP. Tissue-non-specific alkaline phosphatase (TNSALP) is a lipid-anchored ectophosphatase present on the external surface of the cell membrane in several organs such as liver, bone and kidney. Its physiological role is to dephosphorylate B_6_ vitamers so as to allow their transport across the membrane [54]. On the other hand, an intracellular, cytosolic PLP phosphatase exists, which is ubiquitously expressed in humans and is specifically involved in vitamin B_6_ catabolism [55].

Another important component of vitamin B_6_ metabolism is the recently discovered PLP-binding protein (PLP-BP), widespread in all kingdoms of life, with no catalytic activity but with an important regulatory function in PLP homeostasis [56]. In fact, PLP is a very reactive aldehyde that easily combines with amino and thiol groups. Therefore, it is important to maintain a correct balance among B_6_ vitamers inside the cell and keep intracellular-free PLP concentration below toxic levels, but enough to saturate all PLP-dependent enzymes [57].

B_6_ vitamers are absorbed from food and from the intestinal microflora. The richest sources of vitamin B_6_ include fish, beef liver and other organ meats, potatoes and other starchy vegetables, and fruit. In animal-derived foods, vitamin B_6_ is mainly present as PLP, associated with glycogen phosphorylase, and in smaller amounts as PMP, while in plants it is present as PN and PN-5′-β-d-glucoside [58]. Once ingested, PLP, PNP and PMP are dephosphorylated by the ecto-enzyme TNSALP. PM, PN and PL are absorbed from the upper small intestine by a carrier-mediated system and delivered through the portal circulation to the liver. In this organ, they are converted to PLP through the combined action of PDXK and PNPO. From the liver, PLP bound to albumin and dephosphorylated B_6_ vitamers reach all tissues through the blood stream. In order to enter the cells, PLP needs to be dephosphorylated again by membrane-associated TNSALP. Membrane transporters of B_6_ vitamers are yet to be identified. In the cytoplasm, PL, PN and PM are converted into the 5′-phosphorylated vitamers by PDXK, while PNPO converts PNP and PMP into PLP [59]. Once made available, PLP is somehow targeted to the dozens of different apo-B_6_ enzymes that are being synthesized in the cell. Catabolism of vitamin B_6_ consists in the oxidation of PL to 4-pyridoxic acid by aldehyde oxidase 1 (AOX-1) and NAD-dependent dehydrogenases [60].

### 2.2. Effects of vitamin B_6_ homeostasis imbalance

The recommended dietary allowance of vitamin B_6_ is less than 2 mg, an amount easily acquired in developed countries within any diet. PLP concentrations tend to be low in people with alcohol dependence [61], obese individuals [62] and pregnant women [63]. Some pathological conditions are associated with vitamin B_6_ deficiency: end-stage renal diseases, chronic renal insufficiency and other kidney diseases [63]. In addition, vitamin B_6_ deficiency can result from malabsorption syndromes, such as celiac disease, inflammatory bowel diseases including Crohn's disease and ulcerative colitis [63,64]. Certain genetic diseases, such as homocystinuria, can also cause vitamin B_6_ deficiency [65]. People with rheumatoid arthritis often have low vitamin B_6_ concentrations, and vitamin B_6_ concentrations tend to decrease with increased disease severity [66]. Moreover, the assumption of certain drugs, such as contraceptives, and natural compounds may reduce PLP availability [67,68]. The symptoms of PLP deficiency determined by the above-mentioned conditions can be reverted by vitamin B_6_ supplementation. It is known that vitamin B_6_ supplements can also reduce the symptoms of premenstrual syndrome [69], and are used to treat nausea and vomiting in pregnancy [70] as well as carpal tunnel syndrome [71]. Unfortunately, about 28–36% of the general population uses supplements containing vitamin B_6_, even when unnecessary. It is important to maintain the correct balance of vitamin B_6_ because several reports indicated that its excess is neurotoxic. Large doses of vitamin B_6_ have detrimental effects (when the intake exceeds 200 mg day^−1^), mostly evident at the level of the peripheral nervous system [59].

Importantly, perturbations of PLP homeostasis can also have genetic origins, determined by mutations in genes encoding proteins involved in vitamin B_6_ metabolism, and causing severe neurological conditions (table 3). However, increasing evidence is accumulating that vitamin B_6_ deficiency can also contribute to or be the main cause of the onset of serious diseases such as cancer and diabetes, as will be discussed in the following paragraphs.

**Table 3.** Inheritable diseases caused by PLP deficiency.

name of disease and OMIM entry	gene involved in disease and name of encoded protein	affected metabolism	symptoms	available treatments	bibliography
PNPO deficiency (OMIM 610090)	*PNPO*; pyridoxine 5′-phosphate oxidase	PLP salvage pathway	severe neonatal/infantile seizures; few cases with onset after first year of life	pyridoxine/PLP supplementation	[72–77]
alkaline phosphatase deficiency (hypophosphatasia) according to age of onset: adult (OMIM 146300); perinatal (OMIM 241500); infantile (OMIM241500); childhood (OMIM 241510); odontohypophosphatasia (OMIM 146300)	*ALPL*; tissue-non-specific alkaline phosphatase	cellular uptake of B_6_ vitamers	defective mineralization of bone and teeth; wide clinical spectrum, from stillbirth to fractures of the lower extremities or even no bone manifestations (odontohypophosphatasia)	pyridoxine/pyridoxal supplementation	[78–86]
hereditary motor and sensory neuropathy, type VIC, with optic atrophy (OMIM 618511)	*PDXK*; pyridoxal kinase	PLP salvage pathway	progressive distal muscle weakness and atrophy of lower limbs; onset of neuropathy in the first decade, with difficulty of walking and running, followed by similar involvement of upper limbs and hands; distal sensory impairment; progressive optic atrophy and visual impairment during adulthood	PLP supplementation	[87]
PLP-binding protein deficiency (early-onset vitamin B6-dependent epilepsy) (OMIM 617290)	*PLPBP;* (pyridoxal phosphate-binding protein)	intracellular homeostatic regulation of PLP	onset of seizures in the neonatal period or first months of life	pyridoxine/PLP supplementation	[56]
pyridoxine-dependent epilepsy (α-aminoadipic semialdehyde dehydrogenase or antiquitin deficiency) (OMIM 266100)	*ALDH7A1* (α-aminoadipic semialdehyde dehydrogenase or antiquitin)	lysine degradation pathway; accumulation of pipecolic acid in plasma and cerebrospinal fluid	recurrent seizures in the prenatal, neonatal and postnatal period; few cases with onset after first year of life and adolescence	pyridoxine supplementation	[88–91]
L-Δ^1^-pyrroline-5-carboxylate dehydrogenase deficiency (Hyperprolinaemia type II) (OMIM 239510)	*ALDH4A1* (L-Δ^1^-pyrroline-5-carboxylate dehydrogenase)	proline degradation pathway; accumulation of proline and L-Δ^1^-pyrroline-5-carboxylic acid in plasma	often benign but clinical signs may include neonatal/infantile seizures; onset of seizures usually in infancy or childhood	pyridoxine supplementation	[92–95]

## 3. Relationships between vitamin B_6_, DNA damage and cancer inferred by epidemiological studies

### 3.1. Antioxidant properties of vitamin B_6_

The antioxidant properties of B_6_ vitamers were first recognized when it was discovered that the biosynthesis of vitamin B_6_ is essential for the resistance of *Cercospora nicotianae* to singlet-oxygen-generating phototoxins [41]. The efficient activity of vitamin B_6_ in quenching reactive oxygen species (ROS) was also demonstrated in plants [96]. Reduced levels of vitamin B_6_ were associated with severe susceptibility to abiotic stress (oxidative, salt, drought, UVB) in plants, fungi and yeast [97]. Several studies demonstrated that the antioxidant properties of B_6_ vitamers can derive from their direct involvement in reactions with ROS [42,98,99]. The strong antioxidant activity of B_6_ vitamers originates from the presence of both hydroxyl (–OH) and amine (–NH_2_) substituents on the pyridine ring, which can directly react with the peroxy radicals [100]. The antioxidant properties of vitamin B_6_ also have an indirect cause and are surely linked to its role as enzyme cofactor. Studies on the radical-mediated oxidative damage in human whole blood demonstrated a surprising antioxidant activity of pyridoxine [101]. These observations may be attributed to the role of PLP as cofactor in the transulfuration pathway, in which homocysteine is converted to cysteine, a precursor of glutathione, a key regulator of intracellular redox state. It is known that vitamin B_6_ and FD can lead to elevated homocysteine levels, which in turn generate ROS [102]. Also, the gasotransmitter H_2_S and taurine, involved in inflammation and chronic illnesses, derive from sulfur amino acids through the action of PLP-dependent enzymes [103]. The antioxidant properties of vitamin B_6_ are also likely to be connected to its recognized role as anti-inflammatory agent, although a clear link between inflammation, B_6_ status and carcinogenesis has not yet been established [103]. On the other hand, it has been demonstrated that B_6_ vitamers are endogenous photosensitizers that enhance UVA-induced photooxidative stress in human skin. In particular, PL is the most phototoxic UVA-activated vitamer, probably because of the excited triplet state photochemistry associated with its aldehyde group [104].

### 3.2. Vitamin B_6_ availability and cancer risk

The antioxidant properties of vitamin B_6_ are expected to be beneficial in terms of cancer prevention and therapy. However, vitamin B_6_ supplementation has been found to have controversial effects on tumour insurgence and progression [105]. In the attempt to interpret such controversial behaviour, it should be considered that PLP, being involved as cofactor in several biosynthetic pathways, is required for cell proliferation. Therefore, the availability of vitamin B_6_ is bound to affect oncogenesis and tumour progression. Under this perspective, until the early 1980s, restricting vitamin B_6_ availability was considered a promising therapeutic approach against cancer [105]. However, analyses performed on a large number of observations gave evidence of a strong inverse association between both vitamin B_6_ dietary intake and PLP blood levels and cancer [106,107]. Vitamin B_6_ deficiency is linked to a clear increase of several types of tumours, in particular affecting the gastrointestinal tract [108,109] and lungs [107]. Therefore, it is clear that vitamin B_6_ has a complex and multifaceted role in cancer, as an antioxidant preventive agent, but also as an essential micronutrient required for cell proliferation. The low vitamin B_6_ levels observed in cancer patients may be linked to the increased biosynthetic requirements of tumour cells and may also be partially responsible for their decreased immunity.

### 3.3. Expression of vitamin B_6_ metabolism genes and cancer

Referring to the role of vitamin B_6_ in cell proliferation, there is very strong evidence of an association between the expression of genes involved in the recycling of PLP and cancer.

The PNPO gene, encoding pyridoxine 5′-phosphate oxidase, is one out of seven genes, selected among 6487, whose altered expression was found to have a prognostic value in patients with colorectal cancer, and the expression of PNPO is increased in colorectal cancer tissues compared with adjacent normal tissues [110]. This is a clear evidence of the involvement of vitamin B_6_ metabolism in cancer. Several other evidences have been reported, showing a link between salvage pathway enzymes and different kinds of tumours. Zhang *et al.* [111] demonstrated that PNPO contributes to the progression of ovarian surface epithelial tumours. Also in this case, PNPO was found to be overexpressed, and its knockdown induced cell apoptosis and decreased cell proliferation, migration and invasion *in vitro*. Moreover, silencing of PNPO inhibited tumour formation *in vivo* in orthotopically implanted nude mice. Interestingly, the same work also suggested that PLP is important for the regulation of PNPO expression, because PLP supplementation had the effect to suppress PNPO protein expression, resulting in the inhibition of epithelial ovarian cell proliferation. Furthermore, PNPO expression was shown to be regulated by the transforming growth factor-β, probably through the upregulation of a small RNA (miR-143-3p). PNPO is overexpressed also in breast invasive ductal carcinoma, where the expression level is inversely correlated with the overall survival of patients [112]. Moreover, knockdown of PNPO results in a decrease of breast cancer cell proliferation, migration, invasion and colony formation, arrests cell cycle at the G2/M phase and induces cell apoptosis. Also in this case, non-coding RNAs (MALAT1 and mir-216b-5p) are involved in PNPO regulation [112].

PDXK was demonstrated to be upregulated in non-small-cell lung cancer (NSCLC) [113]. Because the high protein levels of PDXK in the tumour did not correlate with the amounts of its mRNA, the authors suggested that PDXK expression is subjected to a post-translational control. Analogously, PDXK was recently found to be abundantly expressed in myeloid leukaemia cells, where PDXK depletion has an antiproliferative effect, which neither PN nor PM was able to rescue [114]. Consistently, pharmacological inhibition of PDXK using isoniazid or the more specific 4′-O-methylpyridoxine (gingkotoxin) has the same effect as genetic depletion of PDXK, suggesting that vitamin B_6_ in plasma supports leukaemia proliferation [114]. On the other hand, it has been demonstrated that PDXK knockdown in human NSCLC cells protects against the cytotoxic activity of different agents, in particular the chemotherapy agent cisplatin, whereas PN administration improved, in a manner that depends on the presence of PDXK, cisplatin anti-tumour effect by exacerbating DNA damage. These observations were attributed to a pharmacokinetic effect of vitamin B_6_, which favours the intracellular accumulation of cisplatin [115].

**Figure 3.**
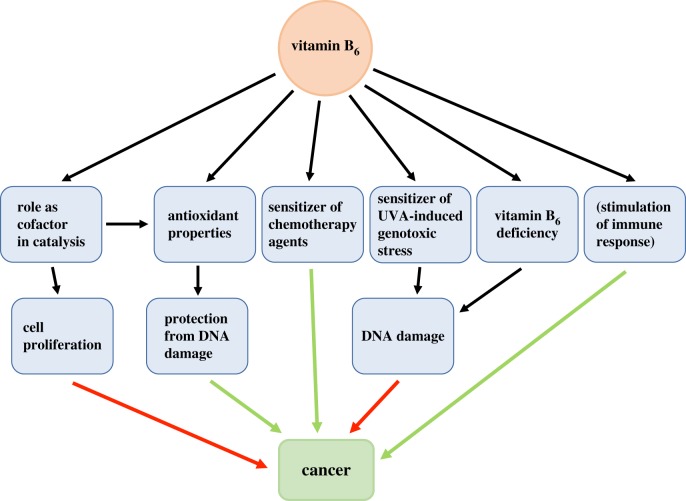
Relationships between vitamin B_6_ and cancer. In the scheme, green arrows represent a protective effect against cancer, whereas red arrows indicate a promoting cancer effect.

Taken together, these data suggest that the effect of vitamin B_6_ on cancer should be examined from different points of view, according to the particular context that is taken under consideration, such as the protection from DNA damage, stimulation of immune response or cell proliferation (figure 3).

### 3.4. Impact of vitamin B_6_ deficiency on DNA metabolism

Expectedly, vitamin B_6_ deficiency plays an important role in DNA damage and repair. PLP is the cofactor of serine hydroxymethyltransferase (SHMT), whose folate-dependent reaction is the main source of one carbon units in metabolism, and plays a fundamental role in the synthesis of thymidylate (figure 1). PLP deficiency may determine a decrease in activity of SHMT, thereby causing the misincorporation of uracil in DNA [116–118]. Another enzyme that depends on both PLP and folate for its activity, glycine decarboxylase, a subunit of the glycine cleavage system, is fundamental for the synthesis of purines and therefore for DNA metabolism [119].

Given the implication of vitamin B_6_ in DNA metabolism, it is not surprising that low vitamin B_6_ levels have been associated with the formation of micronuclei in animal models [120] and in patients affected by inflammatory bowel disease [121]. Vitamin B_6_ can impact on DNA also through different mechanisms. It has been proposed that vitamin B_6_ suppresses endothelial cell proliferation and angiogenesis by inhibiting the activities of DNA polymerase and DNA topoisomerases [122]. In addition, in epatoma cells, PL induces the expression of the insulin-like growth factor-binding protein 1 via a mechanism involving the ERK/c-Jun pathway [123]. Moreover, the same vitamer was shown to play a role in increasing the expression of p21 via p53 activation in several cancer cells and mouse colon [124].

The proper uptake of vitamins is crucial to maintain genome stability, but it is important also to take into consideration the impact that an individual's genotype can have on the capacity to absorb, transport and metabolize vitamins. This could affect the intracellular level of vitamin B_6_, which does not necessarily correspond to the level and distribution of plasmatic vitamin B_6_.

An emerging body of research is focused on understanding how the genome affects folate metabolism and disease risk. The study of common polymorphisms in genes encoding for proteins required for folate metabolism (e.g. methylenetetrahydrofolate reductase (MTHFR; 677C > T), MS (MTR; 2756 A > G)) and uptake (e.g. glutamate carboxypeptidase II (GCPII; 1561 C > T), reduced folate carrier (RFC; 80 G > A)) revealed altered catalytic activity or expression of these proteins, suggesting that they can have a critical impact on developmental or progression of diseases [125,126]. Furthermore, since some of these enzymes for their function require other dietary cofactors (e.g. vitamin B_2_ and B_12_ are cofactors for MTHFR and MTR, respectively), it is important to consider not only nutrient-gene interactions but also interactions of folate with other nutrients. However, these kinds of studies performed *in vitro* or through human genetic screening suffer from the limitations imposed by these complex systems. To overcome these difficulties, the genetic approach applied to model organisms represents the best choice as it allows one to evaluate in whole organisms the phenotypic consequences elicited by mutations in genes involved in metabolism of specific vitamins. In this review, we show how this approach was successful in *Drosophila,* by providing novel approaches for determining the molecular mechanisms correlating micronutrients imbalance and cancer.

## 4. *Drosophila* as a model system to study the effects of B_6_ depletion

Model organisms offer suitable contexts to study the physiopathology of many human diseases by overcoming the difficulties associated with human research. In this contest, *Drosophila melanogaster* has several advantages including an affordable maintenance, a short life cycle, a high fecundity, a relatively brief generation time, a well-characterized genome, a manageable number of chromosomes (consisting in a pair of sex chromosomes along with three pairs of autosomes) and the availability of several mutant fly lines. Main metabolic molecular pathways are well conserved and about 75% of known human disease genes have related sequences in *Drosophila*. Thus, hypotheses and models generated using flies often prove to be relevant to biomedicine. In the last few years, *Drosophila* has been considered a precious model for several metabolic diseases including diabetes and has acquired interest for nutritional intervention studies. The impacts of diet on lifespan, locomotor activity, intestinal barrier function and gut microbiota, as well as fertility, have been evaluated in order to investigate diet-induced pathophysiological mechanisms including inflammation and stress response [127]. However, so far, only a few papers in the literature report studies on the role of vitamins in *Drosophila*. Bahadorani *et al.* [128] studied antioxidant and pro-oxidant properties of vitamin A, C and E to *Drosophila* lifespan under normoxia and oxidative stress. Other investigations [129,130] focused on the role of fly microbiota in providing essential folates and vitamin B_1_ to their host when those are scarce in the diet. Another study [131] showed that folate supplementation was able to alleviate mitochondrial dysfunction in a Parkinson fly model. However, to our knowledge, only vitamin B_6_ has been studied in detail in a *Drosophila* model with the aim to understand cellular and molecular mechanisms at the basis of its beneficial effect on human diseases [132–135].

### 4.1. Impact of mutations in *PDXK* and *PNPO* genes, involved in vitamin B_6_ activation, on genome stability

Like mammals, *Drosophila* produces PLP through the salvage pathway, by recycling precursors from food. In the standard food where flies grow, vitamin B_6_ is present in brewer's yeast, which is rich in PM and PN but poor in PL [136]. As a consequence, it is expected that PDXK and PNPO depletion shall cause similar phenotypes by blocking PLP synthesis.

Molecular mechanisms at the basis of vitamin B_6_ metabolic functions have been investigated in detail by examining phenotypes elicited by mutations (*dPdxk^1^* and *dPdxk^2^*) in PDXK encoding gene. Cytological analysis revealed 5% of chromosome aberrations (CABs) in larval brain cells from *dPdxk* mutants (in wild-type brains, CABs frequency ranges from 0.3 to 0.5% [137]). CABs, such as chromatid deletions, isochromatid deletions and chromosome exchanges, were completely rescued by PLP supplementation (1 mM), suggesting that PLP is important for chromosome integrity maintenance. As a confirmation of this, wild-type larvae treated with PLP inhibitors, such as 4-deoxypiridoxine (4-DP), cycloserine, penicyllamine and isoniazid, also displayed high CAB frequency [132]. Interestingly, most of these compounds are currently used as drugs for several human diseases, such as depression, arthritis and tuberculosis, which have the side effect of decreasing PLP levels [65].

In *Drosophila*, the counterpart of PNPO enzyme is encoded by *Sgll* gene [138]. The depletion of this enzyme causes epilepsy in human, *Drosophila* and zebrafish [139–141]. Silencing of *Sgll* by RNA interference produced 3% of CABs, and was rescued not only by PLP but also by PL supplementation [135].

Data obtained in *Drosophila* are in line with those obtained in yeast, in which it has been demonstrated that mutations in *BUD16* gene (the gene encoding PDXK in yeast) induce gross chromosome rearrangements [142]. Furthermore, also in human cells, PDXK depletion and 4-DP treatment in mock cells cause CABs [132] and 53BP1 repair foci [142], confirming the role of PLP in genome integrity maintenance also in higher organisms, consistently with the presence of micronuclei found in cells with low B_6_ levels [120,121].

### 4.2. Mechanisms through which vitamin B_6_ protects from DNA damage

Vitamin B_6_, as well as folate and vitamin B_12_, are involved in the one carbon metabolism, a crucial pathway for DNA synthesis and repair (figure 1). In particular, vitamin B_6_ serves as coenzyme for the activity of SHMT, which directs one carbon units towards thymidylate synthesis. HPLC analysis revealed that *dPdxk* mutants display increased dUTP/dTTP ratio, but they do not show increased sensitivity to hydroxyurea (HU), a drug which interferes with replication process [132]. This suggests that replication failure is not at the basis of the CABs in *dPdxk* mutants. However, as nucleotide imbalance also affects DNA repair, it is possible that it may contribute to CABs. By contrast, in yeast, HU dramatically affects the growth of *bud16*Δ mutant cells, thus it has been hypothesized that PLP deficiency triggers DNA lesions due to a nucleotide imbalance [142].

**Figure 4.**
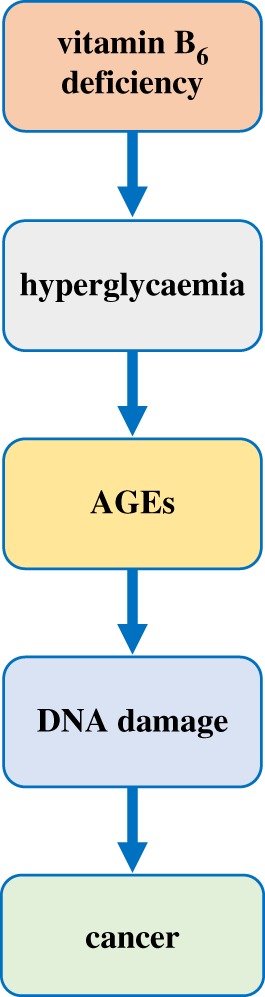
Effects of vitamin B_6_ deficiency inferred from studies carried out in *Drosophila*.

Studies in *Drosophila* revealed that hyperglycaemia induced by low PLP levels might represent another potential cause of CABs. In fact, besides CABs, *dPdxk* mutants display increased glucose content in larval haemolymph in part due to insulin resistance [132], a metabolic condition at the basis of type 2 diabetes. Diabetic hallmarks are also evident in flies fed with 4-DP and in flies depleted of Sgll, which also exhibit impaired lipid metabolism and small body size, a typical feature of diabetic flies [133,135]. The hypothesis that high glucose can produce CABs in low PLP contexts came from two observations. *dPdxk* mutants, Sgll-depleted larvae and 4-DP-fed larvae grown in a medium supplemented with sugars (sucrose or glucose or fructose) exhibit a further increase in CABs (ranging from 15 to 60%), differently from wild-type larvae in which sugar treatment leaves unchanged CAB frequency [132,135]. In addition, *dPdxk* mutants, Sgll-depleted flies and 4-DP-fed larvae accumulated high concentrations of advanced glycation end products (AGEs) in brains [132,133,135]. In high-glucose conditions, these molecules originate from non-enzymatic glycation of amino groups of proteins and DNA and are genotoxic due to ROS formation [143]. AGEs have been associated with diabetic complications and are quenched by PLP and PM [51,52]. Interestingly, α-lipoic acid, a compound able to decrease AGE formation, rescues not only AGEs but also CABs in brains from *dPdxk^1^* mutants, Sgll-depleted individuals and 4-DP-fed larvae [132,135]. Taken together, these findings suggested that in low PLP conditions, CABs are mostly produced by hyperglycaemia, which in turn promotes AGE accumulation that causes DNA damage [132,135] (figure 4). Studies on human cells confirmed this model, as in HeLa cells depleted for PDXK enzyme glucose treatment increases CABs and lipoic acid is effective in rescuing them [132]. Interestingly, a combined effect of low vitamin levels and high glucose in inducing DNA damage has also been found for folates in human cell lines [144].

As mentioned above, some studies indicated the existence of a correlation between low PLP levels and cancer. Although mechanisms behind this association are not clear, it has been hypothesized that low PLP levels can impact on cancer through different mechanisms, for example, by increasing inflammation, decreasing immune defences and promoting genome instability [105]. The finding obtained in *Drosophila* not only confirmed the hypothesis that low PLP levels increase cancer risk through DNA damage, but also revealed that DNA damage in PLP-deficient cells can in part be due to AGE accumulation, which adds to our knowledge of the complex relationship between vitamin B_6_ and cancer.

### 4.3. Vitamin B_6_ as a potential link between diabetes and cancer

Recent data obtained in *Drosophila* suggested that low PLP levels may increase cancer risk in diabetic patients, providing a mechanistic link between studies in humans that associate PLP with cancer and studies indicating that diabetic patients have a higher risk of developing various types of cancer [145–147]. It has been shown that treatment with vitamin B_6_ antagonist 4-DP resulted in much more severe DNA damage in diabetic individuals than in wild-type flies. Brains from two different models of type 2 diabetes displayed 60–80% of CABs (versus 25% in wild-type) and accumulated many more AGEs. Moreover, double mutants bearing *dPdxk^1^* mutation which abolishes PLP production and *Akt1^04226^* mutation which impairs insulin signalling showed a synergistic interaction in CABs formation [133]. It is well known that diabetic condition increases oxidative stress and impairs DNA repair [148]. Accordingly, oxidative damage and DNA strand breaks have been found in both type 1 and type 2 diabetic patients [149,150]. Thus, in a diabetic context, PLP deficiency enhances genome instability by producing a further weakening of antioxidant defence and enhancing hyperglycaemia, contributing to DNA damage throughout ROS induced by AGEs. Since CABs are strictly linked to cancer development and/or progression, extrapolated to humans, these data indicate that low PLP levels may represent a cancer risk factor for diabetic patients. This finding is particularly relevant because the diabetic condition *per se* lowers PLP levels in animal models and patients [151]. Moreover, these data reinforce the hypothesis that besides inflammation, hyperinsulinaemia and hyperglycaemia, DNA damage plays an important role in driving diabetic cells towards malignant transformation.

### 4.4. Validation in *Drosophila* of PDXK human variants and their impact on chromosome integrity

*Drosophila* is also a useful means of validating the causative nature of candidate genetic variants found in patients, and of obtaining functional information on the relationship between disease and linked gene [152]. This approach has been employed to further confirm the role of *PDXK* human gene in chromosome integrity maintenance and to strengthen the model in which CABs are largely produced by hyperglycaemia in low PLP conditions [134]. From these studies it emerged that the expression in *dPdxk^1^* flies of four PDXK variants (three—D87H, V128I and H246Q—listed in databases, and one—A243G—found in a genetic screening in patients with diabetes) was unable to rescue CABs, hyperglycaemia and AGE accumulation, differently from PDXK wild-type protein. Moreover, biochemical analysis of D87H, V128I, H246Q and A243G mutant proteins revealed reduced catalytic activity and reduced affinity for B_6_ vitamers, giving an explanation for this behaviour. Although these variants are rare in population and carried in heterozygous condition, these findings suggest that in certain metabolic contexts and diseases in which PLP levels are reduced, the presence of these PDXK variants could threaten genome integrity and contribute to increased cancer risk.

## 5. Conclusion

B group vitamins are crucial compounds for human health, as they have a strong impact on genome stability and cancer. The relationship between vitamin B_6_ and cancer, deduced from studies reported in this review, is complex and leads us to speculate that it can result from a balance between its antioxidant properties on the one hand and its role as a micronutrient important for cell metabolism on the other hand.

As described in this review, *D. melanogaster* turned out to be a precious model for this kind of study. Findings obtained in *Drosophila* provided information regarding the mechanisms at the basis of the impact of vitamin B_6_ on DNA damage, revealing that AGEs can play an important role. In addition, they suggest that low vitamin B_6_ levels could represent a cancer risk factor in diabetes patients. Future studies in this model organism will be useful to further deepen knowledge of the mechanisms by which vitamin B_6_ and other vitamins can protect against DNA damage and cancer, with the aim of developing personalized treatments.

## Supplementary Material

Reviewer comments

## Data accessibility

This article does not contain any additional data.

## References

[1] Fenech MF 2010 Dietary reference values of individual micronutrients and nutriomes for genome damage prevention: current status and a road map to the future. Am. J. Clin. Nutr. 91, 1438S–1454S. (10.3945/ajcn.2010.28674D) 20219957

[2] Fenech M 2005 The Genome Health Clinic and Genome Health Nutrigenomics concepts: diagnosis and nutritional treatment of genome and epigenome damage on an individual basis. Mutagenesis 20, 255–269. (10.1093/mutage/gei040) 15956042

[3] Jacky PB, Beek B, Sutherland GR 1983 Fragile sites in chromosomes: possible model for the study of spontaneous chromosome breakage. Science 220, 69–70. (10.1126/science.6828880) 6828880

[4] Reidy JA, Zhou X, Chen AT 1983 Folic acid and chromosome breakage. I. Implications for genotoxicity studies. Mutat. Res. 122, 217–221. (10.1016/0165-7992(83)90062-3) 6656813

[5] Wang X, Thomas P, Xue J, Fenech M 2004 Folate deficiency induces aneuploidy in human lymphocytes *in vitro*-evidence using cytokinesis-blocked cells and probes specific for chromosomes 17 and 21. Mutat. Res. 551, 167–180. (10.1016/j.mrfmmm.2004.03.008) 15225591

[6] Fenech M 2006 Cytokinesis-block micronucleus assay evolves into a ‘cytome’ assay of chromosomal instability, mitotic dysfunction and cell death. Mutat. Res. 600, 58–66. (10.1016/j.mrfmmm.2006.05.028) 16822529

[7] Fenech M, Baghurst P, Luderer W, Turner J, Record S, Ceppi M, Bonassi S 2005 Low intake of calcium, folate, nicotinic acid, vitamin E, retinol, beta-carotene and high intake of pantothenic acid, biotin and riboflavin are significantly associated with increased genome instability—results from a dietary intake and micronucleus index survey in South Australia. Carcinogenesis 26, 991–999. (10.1093/carcin/bgi042) 15705599

[8] Young SS, Eskenazi B, Marchetti FM, Block G, Wyrobek AJ 2008 The association of folate, zinc and antioxidant intake with sperm aneuploidy in healthy non-smoking men. Hum. Reprod. 23, 1014–1022. (10.1093/humrep/den036) 18353905

[9] Fenech M, Aitken C, Rinaldi J 1998 Folate, vitamin B12, homocysteine status and DNA damage in young Australian adults. Carcinogenesis 19, 1163–1171. (10.1093/carcin/19.7.1163) 9683174

[10] Stopper H, Treutlein AT, Bahner U, Schupp N, Schmid U, Brink A, Perna A, Heidland A 2008 Reduction of the genomic damage level in haemodialysis patients by folic acid and vitamin B12 supplementation. Nephrol. Dial. Transplant. 23, 3272–3279. (10.1093/ndt/gfn254) 18469307

[11] MacFarlane AJ, Behan NA, Field MS, Williams A, Stover PJ, Yauk CL 2015 Dietary folic acid protects against genotoxicity in the red blood cells of mice. Mutat Res. 779, 105–111. (10.1016/j.mrfmmm.2015.06.012) 26177356 PMC5094184

[12] Duthie SJ 2011 Folate and cancer: how DNA damage, repair and methylation impact on colon carcinogenesis. J. Inherit. Metab. Dis. 34, 101–109. (10.1007/s10545-010-9128-0) 20544289

[13] Pieroth R, Paver S, Day S, Lammersfeld C 2018 Folate and its impact on cancer risk. Curr. Nutr. Rep. 7, 70–84. (10.1007/s13668-018-0237-y) 30099693 PMC6132377

[14] Goulian M, Bleile B, Tseng BY 1980 Methotrexate-induced misincorporation of uracil into DNA. Proc. Natl Acad. Sci. USA 77, 1956–1960. (10.1073/pnas.77.4.1956) 6929529 PMC348628

[15] Duthie SJ, Hawdon A 1998 DNA instability (strand breakage, uracil misincorporation, and defective repair) is increased by folic acid depletion in human lymphocytes *in vitro*. FASEB J. 12, 1491–1497. (10.1096/fasebj.12.14.1491) 9806758

[16] Wang X, Fenech M 2003 A comparison of folic acid and 5-methyltetrahydrofolate for prevention of DNA damage and cell death in human lymphocytes *in vitro*. Mutagenesis 18, 81–86. (10.1093/mutage/18.1.81) 12473740

[17] Guo X, Ni J, Zhu Y, Zhou T, Ma X, Xue J, Wang X 2017 Folate deficiency induces mitotic aberrations and chromosomal instability by compromising the spindle assembly checkpoint in cultured human colon cells. Mutagenesis 32, 547–560. (10.1093/mutage/gex030) 29165592

[18] Stabler SP 2013 Clinical practice. Vitamin B12 deficiency. N. Engl. J. Med. 368, 149–160. (10.1056/NEJMcp1113996) 23301732

[19] Nielsen MJ, Rasmussen MR, Andersen CB, Nexo E, Moestrup SK 2012 Vitamin B12 transport from food to the body's cells—a sophisticated, multistep pathway. Nat. Rev. Gastroenterol. Hepatol. 9, 345–354. (10.1038/nrgastro.2012.76) 22547309

[20] Mills JL, Scott JM, Kirke PN, McPartlin JM, Conley MR, Weir DG, Molloy AM, Lee YJ 1996 Homocysteine and neural tube defects. J. Nutr. 126, 756S–760S. (10.1016/s0140-6736(95)90165-5) 8598561

[21] Sears DA, Udden MM 2012 Howell-Jolly bodies: a brief historical review. Am. J. Med. Sci. 343, 407–409. (10.1097/MAJ.0b013e31823020d1) 21946828

[22] Fenech MF, Dreosti IE, Rinaldi JR 1997 Folate, vitamin B12, homocysteine status and chromosome damage rate in lymphocytes of older men. Carcinogenesis 18, 1329–1336. (10.1093/carcin/18.7.1329) 9230275

[23] Minnet C, Koc A, Aycicek A, Kocyigit A 2011 Vitamin B12 treatment reduces mononuclear DNA damage. Paediatr. Int. 53, 1023–1027. (10.1111/j.1442-200X.2011.03448.x) 21883683

[24] Palmer AM, Kamynina E, Field MS, Stover PJ 2017 Folate rescues vitamin B_12_ depletion-induced inhibition of nuclear thymidylate biosynthesis and genome instability. Proc. Natl Acad. Sci. USA 114, E4095–EE102. (10.1073/pnas.1619582114) 28461497 PMC5441772

[25] Fenech M 1999 Micronucleus frequency in human lymphocytes is related to plasma vitamin B12 and homocysteine. Mutat. Res. 428, 299–304. (10.1016/S1383-5742(99)00056-3) 10518002

[26] Ni J, Liang Z, Zhou T, Cao N, Xia X, Wang X 2012 A decreased micronucleus frequency in human lymphocytes after folate and vitamin B_12_ intervention: a preliminary study in a Yunnan population. Int. J. Vitam. Nutr. Res. 82, 374–382. (10.1024/0300-9831/a000134) 23823922

[27] Fenech M 2001 The role of folic acid and Vitamin B12 in genomic stability of human cells. Mutat. Res. 475, 57–67. (10.1016/S0027-5107(01)00079-3) 11295154

[28] Gavars D, Perminov D, Tauckels E, Lindenberga I, Auce A, Lejniece S 2019 Association of elevated vitamin B_12_ with oncohematological diseases in a cohort of 79 524 patients from Latvia. Exp. Oncol. 41, 357–362. (10.32471/exp-oncology.2312-8852.vol-41-no-4.13930) 31868326

[29] Gangolf M et al. 2010 Thiamine status in humans and content of phosphorylated thiamine derivatives in biopsies and cultured cells. PLoS ONE 5, e13616 (10.1371/journal.pone.0013616) 21049048 PMC2963613

[30] Zastre JA, Sweet RL, Hanberry BS, Ye S 2013 Linking vitamin B1 with cancer cell metabolism. Cancer Metab. 1, 16 (10.1186/2049-3002-1-16) 24280319 PMC4178204

[31] Boros LG et al. 1997 Oxythiamine and dehydroepiandrosterone inhibit the nonoxidative synthesis of ribose and tumor cell proliferation. Cancer Res. 57, 4242–4248. 9331084

[32] Boros LG 2000 Population thiamine status and varying cancer rates between western, Asian and African countries. Anticancer Res. 20(3B), 2245–2248. 10928186

[33] Richardson AD, Moscow JA 2010 Can an enzyme cofactor be a factor in malignant progression? Cancer biol. Ther. 10, 1112–1114. (10.4161/cbt.10.11.14061) 21099372

[34] van Zaanen HC, van der Lelie J 1992 Thiamine deficiency in hematologic malignant tumors. Cancer 69, 1710–1713. (10.1002/1097-0142(19920401)69:7<1710::AID-CNCR2820690711>3.0.CO;2-D) 1551055

[35] Okai Y, Higashi-Okai K, Sato EF, Konaka R, Inoue M 2007 Potent radical-scavenging activities of thiamin and thiamin diphosphate. J. Clin. Biochem. Nutr. 40, 42–48. (10.3164/jcbn.40.42) 18437212 PMC2291503

[36] Coskun R, Turan MI, Turan IS, Gulapoglu M 2014 The protective effect of thiamine pyrophosphate, but not thiamine, against cardiotoxicity induced with cisplatin in rats. Drug Chem. Toxicol. 37, 290–294. (10.3109/01480545.2013.851688) 24215635

[37] Percudani R, Peracchi A 2003 A genomic overview of pyridoxal-phosphate-dependent enzymes. EMBO Rep. 4, 850–854. (10.1038/sj.embor.embor914) 12949584 PMC1326353

[38] Lambrecht G, Braun K, Damer M, Ganso M, Hildebrandt C, Ullmann H, Kassack M, Nickel P 2002 Structure–activity relationships of suramin and pyridoxal-5'-phosphate derivatives as P2 receptor antagonists. Curr. Pharm. Des. 8, 2371–2399. (10.2174/1381612023392973) 12369951

[39] Dakshinamurti K, Lal KJ, Ganguly PK 1998 Hypertension, calcium channel and pyridoxine (vitamin B6). Mol. Cell. Biochem. 188, 137–148. (10.1023/A:1006832810292) 9823019

[40] Salhany JM, Rauenbuehler PB, Sloan RL 1987 Alterations in pyridoxal 5'-phosphate inhibition of human erythrocyte anion transport associated with osmotic hemolysis and resealing. J. Biol. Chem. 262, 15 974–15 978. 3680238

[41] Ehrenshaft M, Bilski P, Li MY, Chignell CF, Daub ME 1999 A highly conserved sequence is a novel gene involved in de novo vitamin B6 biosynthesis. Proc. Natl Acad. Sci. USA 96, 9374–9378. (10.1073/pnas.96.16.9374) 10430950 PMC17790

[42] Bilski P, Li MY, Ehrenshaft M, Daub ME, Chignell CF 2000 Vitamin B6 (pyridoxine) and its derivatives are efficient singlet oxygen quenchers and potential fungal antioxidants. Photochem. Photobiol. 71, 129–134. (10.1562/0031-8655(2000)071<0129:SIPVBP>2.0.CO;2) 10687384

[43] Dell'Aglio E, Boycheva S, Fitzpatrick TB 2017 The pseudoenzyme PDX1.2 sustains vitamin B6 biosynthesis as a function of heat stress. Plant Physiol. 174, 2098–2112. (10.1104/pp.17.00531) 28550206 PMC5543961

[44] Knockel J, Muller IB, Butzloff S, Bergmann B, Walter RD, Wrenger C 2012 The antioxidative effect of de novo generated vitamin B6 in *Plasmodium falciparum* validated by protein interference. Biochem. J. 443, 397–405. (10.1042/bj20111542) 22242896

[45] Grubman A et al. 2010 Vitamin B(6) is required for full motility and virulence in *Helicobacter pylori*. MBio 1, e00112-10 (10.1128/mBio.00112-10) 21151756 PMC3000542

[46] Dick T, Manjunatha U, Kappes B, Gengenbacher M 2010 Vitamin B6 biosynthesis is essential for survival and virulence of *Mycobacterium tuberculosis*. Mol. Microbiol. 78, 980–988. (10.1111/j.1365-2958.2010.07381.x) 20815826

[47] Xie F, Li G, Wang Y, Zhang Y, Zhou L, Wang C, Liu S, Liu S, Wang C 2017 Pyridoxal phosphate synthases PdxS/PdxT are required for *Actinobacillus pleuropneumoniae* viability, stress tolerance and virulence. PLoS ONE 12, e0176374 (10.1371/journal.pone.0176374) 28448619 PMC5407770

[48] Cellini B, Montioli R, Oppici E, Astegno A, Voltattorni CB 2014 The chaperone role of the pyridoxal 5'-phosphate and its implications for rare diseases involving B6-dependent enzymes. Clin. Biochem. 47, 158–165. (10.1016/j.clinbiochem.2013.11.021) 24355692

[49] Huq MD, Tsai NP, Lin YP, Higgins L, Wei LN 2007 Vitamin B6 conjugation to nuclear corepressor RIP140 and its role in gene regulation. Nat. Chem. Biol. 3, 161–165. (10.1038/nchembio861) 17277785

[50] Tramonti A, Nardella C, di Salvo ML, Pascarella S, Contestabile R 2018 The MocR-like transcription factors: pyridoxal 5'-phosphate-dependent regulators of bacterial metabolism. FEBS J. 285, 3925–3944. (10.1111/febs.14599) 29974999

[51] Nakamura S, Li H, Adijiang A, Pischetsrieder M, Niwa T 2007 Pyridoxal phosphate prevents progression of diabetic nephropathy. Nephrol. Dial. Transplant. 22, 2165–2174. (10.1093/ndt/gfm166) 17449494

[52] Ramis R, Ortega-Castro J, Caballero C, Casasnovas R, Cerrillo A, Vilanova B, Adrover M, Frau J 2019 How does pyridoxamine inhibit the formation of advanced glycation end products? The role of its primary antioxidant activity. Antioxidants (Basel) 8, 344 (10.3390/antiox8090344) PMC6770850 31480509

[53] Nakamura S, Niwa T 2005 Pyridoxal phosphate and hepatocyte growth factor prevent dialysate-induced peritoneal damage. J. Am. Soc. Nephrol. 16, 144–150. (10.1681/ASN.2004020120) 15563557

[54] Said HM 2004 Recent advances in carrier-mediated intestinal absorption of water-soluble vitamins. Annu. Rev. Physiol. 66, 419–446. (10.1146/annurev.physiol.66.032102.144611) 14977409

[55] Jang YM, Kim DW, Kang TC, Won MH, Baek NI, Moon BJ, Choi SY, Kwon O-S 2003 Human pyridoxal phosphatase. Molecular cloning, functional expression, and tissue distribution. J. Biol. Chem. 278, 50 040–50 046. (10.1074/jbc.M309619200) 14522954

[56] Darin N et al. 2016 Mutations in PROSC disrupt cellular pyridoxal phosphate homeostasis and cause vitamin-B6-dependent epilepsy. Am. J. Hum. Genet. 99, 1325–1337. (10.1016/j.ajhg.2016.10.011) 27912044 PMC5142116

[57] di Salvo ML, Contestabile R, Safo MK 2011 Vitamin B(6) salvage enzymes: mechanism, structure and regulation. Biochim. Biophys. Acta 1814, 1597–1608. (10.1016/j.bbapap.2010.12.006) 21182989

[58] McCormick DB 1989 Two interconnected B vitamins: riboflavin and pyridoxine. Physiol. Rev. 69, 1170–1198. (10.1152/physrev.1989.69.4.1170) 2678166

[59] di Salvo ML, Safo MK, Contestabile R 2012 Biomedical aspects of pyridoxal 5'-phosphate availability. Front. Biosci. (Elite Ed) 4, 897–913. (10.2741/e428) 22201923

[60] Stanulovic M, Jeremic V, Leskovac V, Chaykin S 1976 New pathway of conversion of pyridoxal to 4-pyridoxic acid. Enzyme 21, 357–369. (10.1159/000458879) 939227

[61] Cravo ML, Camilo ME 2000 Hyperhomocysteinemia in chronic alcoholism: relations to folic acid and vitamins B(6) and B(12) status. Nutrition 16, 296–302. (10.1016/S0899-9007(99)00297-X) 10758367

[62] Ferro Y, Care I, Mazza E, Provenzano F, Colica C, Torti C, Romeo S, Pujia A, Montalcini T 2017 Protein and vitamin B6 intake are associated with liver steatosis assessed by transient elastography, especially in obese individuals. Clin. Mol. Hepatol. 23, 249–259. (10.3350/cmh.2017.0019) 28750503 PMC5628006

[63] Merrill AH Jr, Henderson JM 1987 Diseases associated with defects in vitamin B6 metabolism or utilization. Annu. Rev. Nutr. 7, 137–156. (10.1146/annurev.nu.07.070187.001033) 3300730

[64] Kowlessar OD, Haeffner LJ, Benson GD 1964 Abnormal tryptophan metabolism in patients with adult celiac disease, with evidence for deficiency of vitamin B6. J. Clin. Invest. 43, 894–903. (10.1172/JCI104975) 14169518 PMC289568

[65] Clayton PT 2006 B6-responsive disorders: a model of vitamin dependency. J. Inherit. Metab. Dis. 29, 317–326. (10.1007/s10545-005-0243-2) 16763894

[66] Chiang EP, Selhub J, Bagley PJ, Dallal G, Roubenoff R 2005 Pyridoxine supplementation corrects vitamin B6 deficiency but does not improve inflammation in patients with rheumatoid arthritis. Arthritis Res. Ther. 7, R1404–R1411. (10.1186/ar1839) 16277693 PMC1297588

[67] Lussana F, Zighetti ML, Bucciarelli P, Cugno M, Cattaneo M 2003 Blood levels of homocysteine, folate, vitamin B6 and B12 in women using oral contraceptives compared to non-users. Thromb. Res. 112, 37–41. (10.1016/j.thromres.2003.11.007) 15013271

[68] Gandhi AK et al. 2012 Crystal structures of human pyridoxal kinase in complex with the neurotoxins, ginkgotoxin and theophylline: insights into pyridoxal kinase inhibition. PLoS ONE 7, e40954 (10.1371/journal.pone.0040954) 22879864 PMC3412620

[69] Wyatt KM, Dimmock PW, Jones PW, Shaughn O'Brien PM 1999 Efficacy of vitamin B-6 in the treatment of premenstrual syndrome: systematic review. BMJ 318, 1375–1381. (10.1136/bmj.318.7195.1375) 10334745 PMC27878

[70] Ebrahimi N, Maltepe C, Einarson A 2010 Optimal management of nausea and vomiting of pregnancy. Int. J. Womens Health 2, 241–248. (10.2147/IJWH.S6794) 21151729 PMC2990891

[71] Ryan-Harshman M, Aldoori W 2007 Carpal tunnel syndrome and vitamin B6. Can. Fam. Physician. 53, 1161–1162. 17872812 PMC1949298

[72] Brautigam C, Hyland K, Wevers R, Sharma R, Wagner L, Stock GJ, Heitmann F, Hoffmann GF 2002 Clinical and laboratory findings in twins with neonatal epileptic encephalopathy mimicking aromatic L-amino acid decarboxylase deficiency. Neuropediatrics 33, 113–117. (10.1055/s-2002-33673) 12200739

[73] Clayton PT, Surtees RA, DeVile C, Hyland K, Heales SJ 2003 Neonatal epileptic encephalopathy. Lancet 361, 1614 (10.1016/S0140-6736(03)13312-0) 12747882

[74] Mills PB et al. 2005 Neonatal epileptic encephalopathy caused by mutations in the PNPO gene encoding pyridox(am)ine 5'-phosphate oxidase. Hum. Mol. Genet. 14, 1077–1086. (10.1093/hmg/ddi120) 15772097

[75] Plecko B et al. 2014 Pyridoxine responsiveness in novel mutations of the PNPO gene. Neurology 82, 1425–1433. (10.1212/WNL.0000000000000344) 24658933 PMC4001193

[76] Ruiz A, Garcia-Villoria J, Ormazabal A, Zschocke J, Fiol M, Navarro-Sastre A, Artuch R, Vilaseca MA, Ribes A 2008 A new fatal case of pyridox(am)ine 5'-phosphate oxidase (PNPO) deficiency. Mol. Genet. Metab. 93, 216–218. (10.1016/j.ymgme.2007.10.003) 18024216

[77] Ware TL, Earl J, Salomons GS, Struys EA, Peters HL, Howell KB, Pitt JJ, Freeman JL 2014 Typical and atypical phenotypes of PNPO deficiency with elevated CSF and plasma pyridoxamine on treatment. Dev. Med. Child Neurol. 56, 498–502. (10.1111/dmcn.12346) 24266778

[78] Iqbal SJ, Brain A, Reynolds TM, Penny M, Holland S 1998 Relationship between serum alkaline phosphatase and pyridoxal-5'-phosphate levels in hypophosphatasia. Clin. Sci. (Lond) 94, 203–206. (10.1042/cs0940203) 9536930

[79] Litmanovitz RO, Dolfin T, Arnon S, Regev R, Grinshpan G, Yamazaki M, Ozono K 2002 Glu274Lys/Gly309Arg mutation of the tissue-nonspecific alkaline phosphatase gene in neonatal hypophosphatasia associated with convulsions. J. Inherit. Metab. Dis. 25, 35–40. (10.1023/A:1015121414782) 11999978

[80] Whyte MP, Mahuren JD, Fedde KN, Cole FS, McCabe ER, Coburn SP 1988 Perinatal hypophosphatasia: tissue levels of vitamin B6 are unremarkable despite markedly increased circulating concentrations of pyridoxal-5'-phosphate. Evidence for an ectoenzyme role for tissue-nonspecific alkaline phosphatase. J. Clin. Invest. 81, 1234–1239. (10.1172/JCI113440) 3350970 PMC329654

[81] Rathbun JC 1948 Hypophosphatasia: a new developmental anomaly. Am. J. Dis. Child 75, 822–831. (10.1001/archpedi.1948.02030020840003) 18110134

[82] Whyte MP, Walkenhorst DA, Fedde KN, Henthorn PS, Hill CS 1996 Hypophosphatasia: levels of bone alkaline phosphatase immunoreactivity in serum reflect disease severity. J. Clin. Endocrinol. Metab. 81, 2142–2148. (10.1210/jcem.81.6.8964842) 8964842

[83] Fedde KN, Michell MP, Henthorn PS, Whyte MP 1996 Aberrant properties of alkaline phosphatase in patient fibroblasts correlate with clinical expressivity in severe forms of hypophosphatasia. J. Clin. Endocrinol. Metab. 81, 2587–2594. (10.1210/jc.81.7.2587) 8675582

[84] Caswell AM, Whyte MP, Russell RG 1991 Hypophosphatasia and the extracellular metabolism of inorganic pyrophosphate: clinical and laboratory aspects. Crit. Rev. Clin. Lab. Sci. 28, 175–232. (10.3109/10408369109106862) 1647780

[85] Heinonen JK 2001 Biological role of inorganic pyrophosphate. Boston, MA: Kluwer Academic Publishers.

[86] Balasubramaniam S, Bowling F, Carpenter K, Earl J, Chaitow J, Pitt J, Mornet E, Sillence D, Ellaway C 2010 Perinatal hypophosphatasia presenting as neonatal epileptic encephalopathy with abnormal neurotransmitter metabolism secondary to reduced co-factor pyridoxal-5'-phosphate availability. J. Inherit. Metab. Dis. 33, 25–33. (10.1007/s10545-009-9012-y) 20049532

[87] Chelban V et al. 2019 PDXK mutations cause polyneuropathy responsive to pyridoxal 5'-phosphate supplementation. Ann. Neurol. 86, 225–240. (10.1002/ana.25524) 31187503 PMC6772106

[88] Hunt AD, Stokes J, McCrory WW, Stroud HH 1954 Pyridoxine dependency: report of a case of intractable convulsions in an infant controlled by pyridoxine. Pediatrics 13, 140–145. 13133562

[89] Plecko B et al. 2005 Pipecolic acid as a diagnostic marker of pyridoxine-dependent epilepsy. Neuropediatrics 36, 200–205. (10.1055/s-2005-865727) 15944906

[90] Mills PB et al. 2006 Mutations in antiquitin in individuals with pyridoxine-dependent seizures. Nat. Med. 12, 307–309. (10.1038/nm1366) 16491085

[91] Scharer G, Brocker C, Vasiliou V, Creadon-Swindell G, Gallagher RC, Spector E, Van Hove JLK 2010 The genotypic and phenotypic spectrum of pyridoxine-dependent epilepsy due to mutations in ALDH7A1. J. Inherit. Metab. Dis. 33, 571–581. (10.1007/s10545-010-9187-2) 20814824 PMC3112356

[92] Emery FA, Goldie L, Stern J 1968 Hyperprolinaemia type 2. J. Ment. Defic. Res. 12, 187–195. 4972625 10.1111/j.1365-2788.1968.tb00258.x

[93] Geraghty MT, Vaughn D, Nicholson AJ, Lin WW, Jimenez-Sanchez G, Obie C, Flynn P, Valle D, Hu CA 1998 Mutations in the Delta1-pyrroline 5-carboxylate dehydrogenase gene cause type II hyperprolinemia. Hum. Mol. Genet. 7, 1411–1415. (10.1093/hmg/7.9.1411) 9700195

[94] Farrant RD, Walker V, Mills GA, Mellor JM, Langley GJ 2001 Pyridoxal phosphate de-activation by pyrroline-5-carboxylic acid. Increased risk of vitamin B6 deficiency and seizures in hyperprolinemia type II. J. Biol. Chem. 276, 15 107–15 116. (10.1074/jbc.M010860200) 11134058

[95] Walker V, Mills GA, Mellor JM, Langley GJ, Farrant RD 2003 A novel pyrroline-5-carboxylic acid and acetoacetic acid adduct in hyperprolinaemia type II. Clin. Chim. Acta 331, 7–17. (10.1016/S0009-8981(03)00077-9) 12691858

[96] Denslow SA, Rueschhoff EE, Daub ME 2007 Regulation of the *Arabidopsis thaliana* vitamin B6 biosynthesis genes by abiotic stress. Plant Physiol. Biochem. 45, 152–161. (10.1016/j.plaphy.2007.01.007) 17344055

[97] Hellmann H, Mooney S 2010 Vitamin B6: a molecule for human health? Molecules 15, 442–459. (10.3390/molecules15010442) 20110903 PMC6257116

[98] Matxain JM, Ristila M, Strid A, Eriksson LA 2006 Theoretical study of the antioxidant properties of pyridoxine. J. Phys. Chem. A 110, 13 068–13 072. (10.1021/jp065115p) 17134167

[99] Ohta BK, Foote CS 2002 Characterization of endoperoxide and hydroperoxide intermediates in the reaction of pyridoxine with singlet oxygen. J. Am. Chem. Soc. 124, 12 064–12 065. (10.1021/ja0205481) 12371824

[100] Kannan K, Jain SK 2004 Effect of vitamin B6 on oxygen radicals, mitochondrial membrane potential, and lipid peroxidation in H_2_O_2_-treated U937 monocytes. Free Radic. Biol. Med. 36, 423–428. (10.1016/j.freeradbiomed.2003.09.012) 14975445

[101] Stocker P, Lesgards JF, Vidal N, Chalier F, Prost M 2003 ESR study of a biological assay on whole blood: antioxidant efficiency of various vitamins. Biochim. Biophys. Acta. 1621, 1–8. (10.1016/S0304-4165(03)00008-4) 12667604

[102] Tinelli C, Di Pino A, Ficulle E, Marcelli S, Feligioni M 2019 Hyperhomocysteinemia as a risk factor and potential nutraceutical target for certain pathologies. Front. Nutr. 6, 49 (10.3389/fnut.2019.00049) 31069230 PMC6491750

[103] Bird RP 2018 The emerging role of vitamin B6 in inflammation and carcinogenesis. Adv. Food Nutr. Res. 83, 151–194. (10.1016/bs.afnr.2017.11.004) 29477221

[104] Justiniano R, Williams JD, Perer J, Hua A, Lesson J, Park SL, Wondrak GT 2017 The B6-vitamer pyridoxal is a sensitizer of UVA-induced genotoxic stress in human primary keratinocytes and reconstructed epidermis. Photochem. Photobiol. 93, 990–998. (10.1111/php.12720) 28083878 PMC5500433

[105] Galluzzi L, Vacchelli E, Michels J, Garcia P, Kepp O, Senovilla L, Vitale I, Kroemer G 2013 Effects of vitamin B6 metabolism on oncogenesis, tumor progression and therapeutic responses. Oncogene 32, 4995–5004. (10.1038/onc.2012.623) 23334322

[106] Mocellin S, Briarava M, Pilati P 2017 Vitamin B6 and cancer risk: a field synopsis and meta-analysis. J. Natl Cancer Inst. 109, 1–9. (10.1093/jnci/djw230) 28376200

[107] Zuo H et al. 2019 Vitamin B6 catabolism and lung cancer risk: results from the Lung Cancer Cohort Consortium (LC3). Ann Oncol. 30, 478–485. (10.1093/annonc/mdz002) 30698666 PMC6442648

[108] Gylling B et al. 2017 Vitamin B-6 and colorectal cancer risk: a prospective population-based study using 3 distinct plasma markers of vitamin B-6 status. Am. J. Clin. Nutr. 105, 897–904. (10.3945/ajcn.116.139337) 28275126

[109] Kayashima T, Tanaka K, Okazaki Y, Matsubara K, Yanaka N, Kato N 2011 Consumption of vitamin B6 reduces colonic damage and protein expression of HSP70 and HO-1, the anti-tumor targets, in rats exposed to 1,2-dimethylhydrazine. Oncol. Lett. 2, 1243–1246. (10.3892/ol.2011.370) 22848295 PMC3406545

[110] Chen H, Sun X, Ge W, Qian Y, Bai R, Zheng S 2017 A seven-gene signature predicts overall survival of patients with colorectal cancer. Oncotarget 8, 95 054–95 065. (10.18632/oncotarget.10982) 29221110 PMC5707004

[111] Zhang L et al. 2017 Pyridoxine 5'-phosphate oxidase is a novel therapeutic target and regulated by the TGF-beta signalling pathway in epithelial ovarian cancer. Cell Death Dis. 8, 3214 (10.1038/s41419-017-0050-3) 29238081 PMC5870590

[112] Ren W, Guan W, Zhang J, Wang F, Xu G 2019 Pyridoxine 5'-phosphate oxidase is correlated with human breast invasive ductal carcinoma development. Aging (Albany, NY) 11, 2151–2176. (10.18632/aging.101908) 30982780 PMC6503878

[113] Galluzzi L et al. 2012 Prognostic impact of vitamin B6 metabolism in lung cancer. Cell Rep. 2, 257–269. (10.1016/j.celrep.2012.06.017) 22854025

[114] Chen CC et al. 2020 Vitamin B6 addiction in acute myeloid leukemia. Cancer Cell. 37, 71–84.e7. (10.1016/j.ccell.2019.12.002) 31935373 PMC7197326

[115] Galluzzi L et al. 2013 Vitamin B6 metabolism influences the intracellular accumulation of cisplatin. Cell Cycle 12, 417–421. (10.4161/cc.23275) 23287530 PMC3587442

[116] MacFarlane AJ, Anderson DD, Flodby P, Perry CA, Allen RH, Stabler SP, Stover PJ 2011 Nuclear localization of de novo thymidylate biosynthesis pathway is required to prevent uracil accumulation in DNA. J. Biol. Chem. 286, 44 015–44 022. (10.1074/jbc.M111.307629) PMC3243516 22057276

[117] Paone A, Marani M, Fiascarelli A, Rinaldo S, Giardina G, Contestabile R, Paiardini A, Cutruzzolà F 2014 SHMT1 knockdown induces apoptosis in lung cancer cells by causing uracil misincorporation. Cell Death Dis. 5, e1525 (10.1038/cddis.2014.482) 25412303 PMC4260740

[118] Giardina G et al. 2018 The catalytic activity of serine hydroxymethyltransferase is essential for de novo nuclear dTMP synthesis in lung cancer cells. FEBS J. 285, 3238–3253. (10.1111/febs.14610) 30035852

[119] Fleming A, Copp AJ 1998 Embryonic folate metabolism and mouse neural tube defects. Science 280, 2107–2109. (10.1126/science.280.5372.2107) 9641914

[120] Almeida MR, Venancio VP, Aissa AF, Darin JDC, Pires Bianchi ML, Greggi Antunes LM 2015 Effects of maternal vitamin B6 deficiency and over-supplementation on DNA damage and oxidative stress in rat dams and their offspring. Food Chem. Toxicol. 80, 201–205. (10.1016/j.fct.2015.03.015) 25818462

[121] Kim SY et al. 2019 Increased genomic damage and vitamin B status in inflammatory bowel disease patients: a case-control, prospective, pilot study. Mutat. Res. Genet. Toxicol. Environ. Mutagen 837, 42–47. (10.1016/j.mrgentox.2018.10.002) 30595208

[122] Matsubara K, Matsumoto H, Mizushina Y, Lee JS, Kato N 2003 Inhibitory effect of pyridoxal 5'-phosphate on endothelial cell proliferation, replicative DNA polymerase and DNA topoisomerase. Int. J. Mol. Med. 12, 51–55. (10.3892/ijmm.12.1.51) 12792808

[123] Zhang P, Suidasari S, Hasegawa T, Yanaka N, Kato N 2013 High concentrations of pyridoxal stimulate the expression of IGFBP1 in HepG2 cells through upregulation of the ERK/cJun pathway. Mol. Med. Rep. 8, 973–978. (10.3892/mmr.2013.1629) 23942851

[124] Zhang P, Suidasari S, Hasegawa T, Yanaka N, Kato N 2014 Vitamin B(6) activates p53 and elevates p21 gene expression in cancer cells and the mouse colon. Oncol. Rep. 31, 2371–2376. (10.3892/or.2014.3073) 24626782

[125] Stover PJ 2011 Polymorphisms in 1-carbon metabolism, epigenetics and folate-related pathologies. J. Nutrigenet. Nutrigenomics 4, 293–305. (10.1159/000334586) 22353665 PMC3696357

[126] Bailey LB et al. 2015 Biomarkers of nutrition for development—folate review. J. Nutr. 145, 1636S–1680S. (10.3945/jn.114.206599) 26451605 PMC4478945

[127] Staats S, Luersen K, Wagner AE, Rimbach G 2018 *Drosophila melanogaster* as a versatile model organism in food and nutrition research. J. Agric. Food Chem. 66, 3737–3753. (10.1021/acs.jafc.7b05900) 29619822

[128] Bahadorani S, Bahadorani P, Phillips JP, Hilliker AJ 2008 The effects of vitamin supplementation on *Drosophila* life span under normoxia and under oxidative stress. J. Gerontol. A Biol. Sci. Med. Sci. 63, 35–42. (10.1093/gerona/63.1.35) 18245758

[129] Blatch SA, Meyer KW, Harrison JF 2010 Effects of dietary folic acid level and symbiotic folate production on fitness and development in the fruit fly *Drosophila melanogaster*. Fly (Austin) 4, 312–319. (10.4161/fly.4.4.13258) 20855945

[130] Dobson AJ, He X, Blanc E, Bolukbasi E, Feseha Y, Yang M, Piper MDW 2018 Tissue-specific transcriptome profiling of *Drosophila* reveals roles for GATA transcription factors in longevity by dietary restriction. NPJ Aging Mech. Dis. 4, 5 (10.1038/s41514-018-0024-4) 29675265 PMC5904217

[131] Srivastav S, Singh SK, Yadav AK, Srikrishna S 2015 Folic acid supplementation rescues anomalies associated with knockdown of parkin in dopaminergic and serotonergic neurons in *Drosophila* model of Parkinson's disease. Biochem. Biophys. Res. Commun. 460, 780–785. (10.1016/j.bbrc.2015.03.106) 25824034

[132] Marzio A, Merigliano C, Gatti M, Verni F 2014 Sugar and chromosome stability: clastogenic effects of sugars in vitamin B6-deficient cells. PLoS Genet. 10, e1004199 (10.1371/journal.pgen.1004199) 24651653 PMC3961173

[133] Merigliano C, Mascolo E, La Torre M, Saggio I, Verni F 2018 Protective role of vitamin B6 (PLP) against DNA damage in *Drosophila* models of type 2 diabetes. Sci. Rep. 8, 11432 (10.1038/s41598-018-29801-z) 30061626 PMC6065437

[134] Mascolo E et al. 2019 The expression of four pyridoxal kinase (PDXK) human variants in *Drosophila* impacts on genome integrity. Sci. Rep. 9, 14188 (10.1038/s41598-019-50673-4) 31578392 PMC6775053

[135] Mascolo E, Amoroso N, Saggio I, Merigliano C, Verni F 2020 Pyridoxine/pyridoxamine 5'-phosphate oxidase (Sgll/PNPO) is important for DNA integrity and glucose homeostasis maintenance in *Drosophila*. J. Cell. Physiol. 235, 504–512. (10.1002/jcp.28990) 31506944

[136] Roth-Maier DA, Kettler SI, Kirchgessner M 2002 Availability of vitamin B6 from different food sources. Int. J. Food Sci. Nutr. 53, 171–179. (10.1080/09637480220132184) 11939111

[137] Merigliano C, Marzio A, Renda F, Somma MP, Gatti M, Verni F 2017 A role for the twins protein phosphatase (PP2A-B55) in the maintenance of *Drosophila* genome integrity. Genetics 205, 1151–1167. (10.1534/genetics.116.192781) 28040742 PMC5340330

[138] Chi W, Zhang L, Du W, Zhuang X 2014 A nutritional conditional lethal mutant due to pyridoxine 5'-phosphate oxidase deficiency in *Drosophila melanogaster*. G3 (Bethesda) 4, 1147–1154. (10.1534/g3.114.011130) 24739647 PMC4065258

[139] Chi W, Iyengar ASR, Albersen M, Bosma M, Verhoeven-Duif NM, Wu CF, Zhuang X 2019 Pyridox (am) ine 5'-phosphate oxidase deficiency induces seizures in *Drosophila melanogaster*. Hum. Mol. Genet. 28, 3126–3136. (10.1093/hmg/ddz143) 31261385 PMC6737294

[140] Wilson MP, Plecko B, Mills PB, Clayton PT 2019 Disorders affecting vitamin B6 metabolism. J. Inherit. Metab. Dis. 42, 629–646. (10.1002/jimd.12030) 30671974

[141] Ciapaite J et al. 2020 Pyridox(am)ine 5'-phosphate oxidase (PNPO) deficiency in zebrafish results in fatal seizures and metabolic aberrations. Biochim. Biophys. Acta Mol. Basis Dis. 1866, 165607 (10.1016/j.bbadis.2019.165607) 31759955

[142] Kanellis P et al. 2007 A screen for suppressors of gross chromosomal rearrangements identifies a conserved role for PLP in preventing DNA lesions. PLoS Genet. 3, e134 (10.1371/journal.pgen.0030134) 17696614 PMC1941753

[143] Booth AA, Khalifah RG, Todd P, Hudson BG 1997 *In vitro* kinetic studies of formation of antigenic advanced glycation end products (AGEs): novel inhibition of post-Amadori glycation pathways. J. Biol. Chem. 272, 5430–5437. (10.1074/jbc.272.9.5430) 9038143

[144] Guo X, Dai X, Ni J, Cao N, Yang G, Xue J, Wang X 2019 High concentration of sugars is genotoxic to folate-deficient cells. Mutat. Res. 814, 15–22. (10.1016/j.mrfmmm.2019.01.003) 30682723

[145] Vigneri R 2009 Diabetes: diabetes therapy and cancer risk. Nat. Rev. Endocrinol. 5, 651–652. (10.1038/nrendo.2009.219) 19924151

[146] Noto H, Tsujimoto T, Sasazuki T, Noda M 2011 Significantly increased risk of cancer in patients with diabetes mellitus: a systematic review and meta-analysis. Endocr. Pract. 17, 616–628. (10.4158/EP10357.RA) 21454235

[147] Dankner R, Boffetta P, Balicer RD, Boker LK, Sadeh M, Berlin A, Olmer L, Goldfracht M, Freedman LS 2016 Time-dependent risk of cancer after a diabetes diagnosis in a cohort of 2.3 million adults. Am J Epidemiology 183, 1098–1106. (10.1093/aje/kwv290) 27257115

[148] Blasiak J, Arabski M, Krupa R, Wozniak K, Zadrozny M, Kasznicki J, Zurawska M, Drzewoski J 2004 DNA damage and repair in type 2 diabetes mellitus. Mutat. Res. 554, 297–304. (10.1016/j.mrfmmm.2004.05.011) 15450427

[149] Goodarzi MT, Navidi AA, Rezaei M, Babahmadi-Rezaei H 2010 Oxidative damage to DNA and lipids: correlation with protein glycation in patients with type 1 diabetes. J. Clin. Lab. Anal. 24, 72–76. (10.1002/jcla.20328) 20333759 PMC6647748

[150] Tatsch E et al. 2012 Association between DNA strand breakage and oxidative, inflammatory and endothelial biomarkers in type 2 diabetes. Mutat. Res. 732, 16–20. (10.1016/j.mrfmmm.2012.01.004) 22285873

[151] Merigliano C, Mascolo E, Burla R, Saggio I, Verni F 2018 The relationship between vitamin B6, diabetes and cancer. Front. Genet. 9, 388 (10.3389/fgene.2018.00388) 30271425 PMC6146109

[152] Oriel C, Lasko P 2018 Recent developments in using *Drosophila* as a model for human genetic disease. Int. J. Mol. Sci. 19, 2041 (10.3390/ijms19072041) PMC6073706 30011838

